# Die Behandlung des Chondroblastoms im Humeruskopf: Eine tumor- und schulterorthopädische Herausforderung

**DOI:** 10.1007/s00132-020-03958-w

**Published:** 2020-08-03

**Authors:** Sebastian Oenning, Kristian Nikolaus Schneider, Georg Gosheger, Christoph Theil, Friederike Müller, Niklas Deventer, Timo Lübben

**Affiliations:** 1 Klinik für Allgemeine Orthopädie und Tumororthopädie, Universitätsklinikum Münster, Albert-Schweitzer-Campus 1, 48149 Münster, Deutschland; 2 Gerhard-Domagk-Institut für Pathologie, Universitätsklinikum Münster, Münster, Deutschland

**Keywords:** Knorpelerkrankung, Kürettage, Fokale Rekonstruktion, Humerus, Schulter, Cartilage diseases, Curettage, Focal resurfacing, Humerus, Shoulder

## Abstract

Wir präsentieren den Fall einer 22-jährigen Patientin mit einem histopathologisch gesicherten Chondroblastom des rechten Humeruskopfes. Um bei der jungen und funktionell anspruchsvollen Patientin eine möglichst schonende und anatomische Rekonstruktion der humeralen Gelenkfläche zu erzielen, ohne dabei auf einen totalendoprothetischen Gelenkersatz zurückzugreifen, implantierten wir nach intraläsionaler Kürettage des Chondroblastoms eine HemiCAP®. Die sehr guten funktionellen Kurzzeitergebnisse zeigen, dass dieser Therapieansatz eine sehr gute Behandlungsoption darstellen kann.

**Video online:**

Die Online-Version dieses Beitrags (10.1007/s00132-020-03958-w) enthält ein Video.

## Abstract (en)

We present the case of a 22-year-old female patient with chondroblastoma in the right humeral head. To allow a gentle and anatomic resurfacing of the humeral joint surface and to avoid total joint arthroplasty in our young patient with high functional requirements, we implanted a HemiCAP® after intralesional curettage of the chondroblastoma. Our patient’s excellent short-term functional outcome shows that our approach can be considered a very good therapeutic option.

## Anamnese

Wir präsentieren den Fall einer 22-jährigen, sportlichen und im Polizeidienst tätigen Patientin (173 cm, 70 kg), die mit einer auswärtig diagnostizierten intraossären Raumforderung im rechten Humeruskopf in unserer Klinik vorstellig wurde.

**Figure 1:**
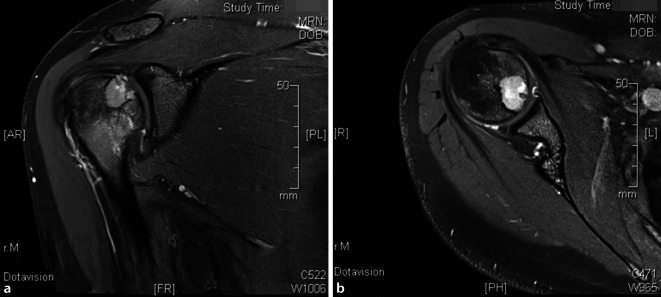

**Figure 2:**
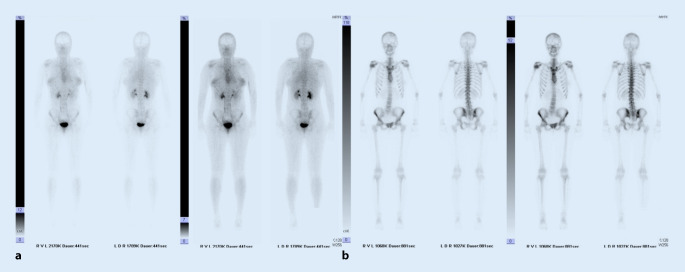

Erstmalige Schulterschmerzen traten 2 Jahre zuvor auf und wurden zunächst frustran konservativ mit nichtsteroidalen Antirheumatika und Physiotherapie behandelt. Eine anschließende MRT-Bildgebung zeigte eine etwa 1,7 cm große, intraossäre Raumforderung des rechten Humeruskopfes (Abb. 1). Ein Staging per Ganzkörper-Dreiphasen-Skelettszintigraphie zeigte einen lokal verstärkten Knochenstoffwechsel ohne weitere signalanreichernde Läsionen (Abb. 2). Ebenfalls auswärtig erfolgte anschließend eine arthroskopische Biopsie, die einen riesenzellreichen Prozess, verdächtig für ein Chondroblastom, ergab.

## Befund und Diagnostik

In der körperlichen Untersuchung zeigte sich eine maximale aktive Abduktion und Anteversion bis 90°. Die Funktionstests der Rotatorenmanschette verblieben allesamt negativ.

Sowohl in der MRT-Bildgebung als auch in der Arthroskopie des Schultergelenks wurde ein erheblicher, fokaler humeraler Knorpeldefekt deutlich (Abb. 1).

**Figure 3:**
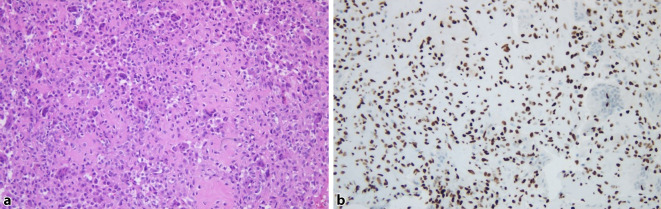

Die referenzpathologische Untersuchung bestätigte den Verdacht auf ein Chondroblastom (Abb. 3).

## Therapie und Verlauf

Ziel unserer Behandlung war es, sowohl den hohen beruflichen Ansprüchen an das Schultergelenk als auch den tumororthopädischen Maßstäben gerecht zu werden. Im Einvernehmen mit der Patientin entschieden wir uns gegen eine transossäre Kürettage und Auffüllung mittels Knochenersatzstoff und für eine transartikuläre Kürettage mit Implantation eines zementierten HemiCAP®-Systems (Arthrosurface, Franklin, MA, USA), um den fokalen Knorpeldefekt zu decken und eine anatomische humerale Gelenkfläche wiederherzustellen. Die Möglichkeit eines vollprothetischen Gelenkersatzes wurde aufgrund des noch jungen Alters und der hohen funktionellen Ansprüche von der Patientin abgelehnt.

**Figure 4:**
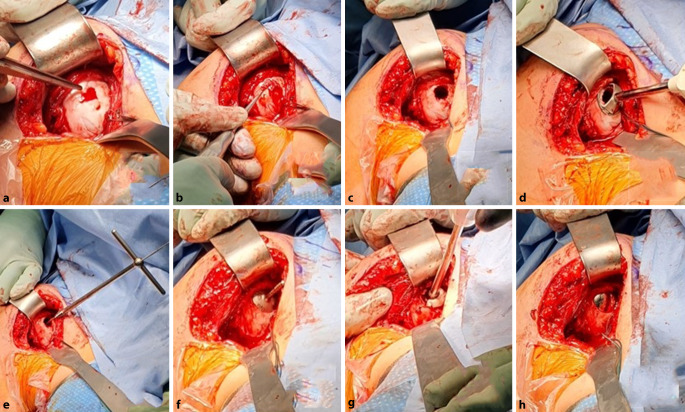

In Allgemeinanästhesie und in Beachchair-Position eröffneten wir über einen deltopektoralen Zugang das Glenohumeralgelenk (Abb. 4). Das Chondroblastom wurde intraläsional kürettiert, der instabile Knorpel in den Randbereichen reseziert und durch einen Probeaufbau wurde die Größe des Implantates ermittelt. Der Knochendefekt wurde mit Polymethylmethacrylat (PMMA) (Palacos, Heraeus Medical, Wehrheim, Deutschland) aufgefüllt, um eine stabile Verankerung der in der Folge eingebrachten Fixationsschraube sowie des HemiCAP®-Implantats (20 mm) zu gewährleisten. Der M. subscapularis wurde mit nichtresorbierbarem Faden refixiert. Intraoperativ zeigte sich ein stabiles Schultergelenk. Die Ruhigstellung erfolgte für 4 Wochen in einer Ultrasling-Orthese (DJO Global, Freiburg, Deutschland).

Histologisch erfolgte eine Diagnosebestätigung und mittels CT-Bildgebung des Thorax wurden Lungenmetastasen ausgeschlossen.

**Figure 5:**
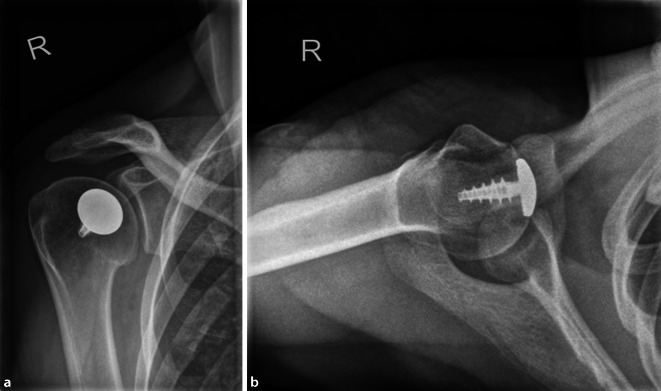

Bei der klinisch-radiologischen Verlaufskontrolle 6 Monate postoperativ zeigte sich eine regelrechte Implantatlage ohne Hinweis auf Lokalrezidiv, Lockerung, Bruch oder Dislokation (Abb. 5). Abduktion und Anteversion waren aktiv bis 170° frei, die Außenrotation betrug rechtsseitig 70°, linksseitig 80° (Video 1). Der American Shoulder and Elbow Surgeons (ASES) Score von 85/100 und der Constant-Score von 81/100 (Gegenseite 90/100) bestätigten das gute funktionelle Ergebnis. Ihre berufliche Tätigkeit kann die Patientin ohne Einschränkungen ausführen.

## Diskussion

Chondroblastome sind benigne Tumoren, die meist im Kindes- oder Jugendalter auftreten. Sie sind meist epiphysär im proximalen Humerus, in der proximalen Tibia sowie im proximalen und distalen Femur lokalisiert [3, 7]. Lokale Rezidivraten variieren in der Literatur von 3–30 % [3, 6, 9]. Ebeid et al. konnten zeigen, dass durch intraläsionale Kürettage und Auffüllen des Defektes mittels Knochenzement oder -graft eine gute lokale Tumorkontrolle erreicht werden kann [3]. Aufgrund der meist gelenknahen Lokalisation von Chondroblastomen ist eine sekundäre Arthrose des betroffenen Gelenkes schon bei jungen Patienten eine mögliche postoperative Komplikation [4]. Zudem beschreiben einzelne Fallstudien bereits im Jugendalter rezidivierende Chondroblastome des Humeruskopfes mit Destruktion der humeralen Gelenkfläche, die mittels Resektion des proximalen Humerus und endoprothetischer Rekonstruktion behandelt wurden [3, 9].

Zur Behandlung fokaler Knorpeldefekte im Bereich des Humeruskopfes hat sich innerhalb der letzten Jahre das HemiCAP®-System etabliert, welches eine anatomisch gelenkerhaltende Rekonstruktion ermöglicht. Generell können die Implantate zur Deckung von beispielsweise degenerativen oder posttraumatischen Knorpelläsionen in allen großen Gelenken angewendet werden. Typische schulterspezifische Indikationen sind vor allem frühe Stadien der Omarthrose oder der Humeruskopfnekrose sowie traumatische Knorpeldefekte [2, 10].

Für die HemiCAP®-Implantation sollten gewisse anatomische Voraussetzungen erfüllt sein. Die Rotatorenmanschette sollte intakt oder reparabel sein, es sollten keine höhergradigen osteophytären Anbauten vorhanden sein, das Glenoid sollte physiologisch geformt und der Humeruskopf regelrecht zentralisiert sein [10]. Diese Voraussetzungen waren bei unserer Patientin gegeben.

Vor allem jüngere, aktive Patienten profitieren von der fokalen Rekonstruktion, die signifikant zu besserer Funktionalität, Schmerzreduktion und hoher Patientenzufriedenheit führt [8, 10]. Verglichen mit Schaftprothesen zeigten sich für fokale Rekonstruktionssysteme bei vergleichbarem Outcome geringere intraoperative Blutverluste sowie eine kürzere Operationsdauer [1]. Gerade bei jungen Patienten sind die Haltbarkeit der Prothese und die operativen Möglichkeiten bei Revisionseingriffen entscheidend. Diesbezüglich konnte gezeigt werden, dass die Revisionsrate bei HemiCAP®-Systemen im Vergleich zu Schaftprothesen geringer ist und zudem mehr Optionen bei Revisionseingriffen bestehen [5, 10].

Bei humeralen, intraartikulären Chondroblastomen kann die Kürettage mit fokaler, anatomischer Rekonstruktion also eine vielversprechende Option zwischen Knochenersatz und Vollendoprothese sein. Langzeitstudien zu Implantatüberleben und etwaigen Komplikationen stehen jedoch aktuell noch aus.

## Fazit für die Praxis

In Zusammenschau zeigt der präsentierte Fall, dass beim intraartikulären Chondroblastom die Kürettage sowie die Implantation einer HemiCAP® eine gute Therapieoption mit guten funktionellen Kurzzeitergebnissen darstellt.

## Caption Electronic Supplementary Material



## Abkürzungen

ASES: American Shoulder and Elbow Surgeons
FS: Fettsättigung
PMMA: Polymethylmethacrylat
TSE: Turbo Spin Echo
